# Lower Leg Compartment Syndrome after Appendicectomy

**DOI:** 10.1155/2015/585986

**Published:** 2015-02-10

**Authors:** Shane C. O'Neill, Darren F. Lui, Colm Murphy, Patrick J. Kiely

**Affiliations:** Department of Paediatric Trauma and Orthopaedics, Our Lady's Children's Hospital Crumlin, Dublin 12, Ireland

## Abstract

A 10-year-old boy presented with severe left lower leg pain, uncontrolled with increasing analgesia after appendicectomy. A diagnosis of acute compartment syndrome was made after a delayed referral to the orthopaedic service. The patient subsequently underwent an emergency fasciotomy and made a good functional recovery. To the best of our knowledge this is the first reported case of paediatric lower leg compartment syndrome after appendicectomy in the literature. The case report serves to highlight the importance of maintaining a high index of suspicion for compartment syndrome.

## 1. Introduction

Acute compartment syndrome is a surgical emergency. It can be defined as a condition in which increased pressure within a limited space compromises the circulation and function of the tissues within that space [1]. It was originally described by von Volkmann in 1882 in relation to a complication after supracondylar fracture [2]. Compartment syndrome in adults is a clinical diagnosis with five major signs described: pain out of proportion, paraesthesia, paralysis, pallor, and pulselessness [3]. Compartment syndrome in the paediatric population has been shown to be more difficult to diagnose and the traditional signs are not as reliable [3]. In children the 3 A's, increasing anxiety, agitation, and increasing analgesic requirements, have been shown to be more predictive of compartment syndrome [3].

Compartment syndrome is most commonly caused by trauma, with supracondylar and tibia fractures most at risk [4]. Other less common aetiologies, such as burns and soft tissue injuries, have also been described [4]. Delayed or missed diagnosis of ACS can result in severe consequences such as tissue necrosis, neurological deficit, limb loss, and even death [4].

We report a case of acute lower leg compartment syndrome after appendicectomy. To the best of our knowledge this is the first such case reported in the literature. The child represented after appendicectomy and had a delayed diagnosis of compartment syndrome. He subsequently underwent emergency fasciotomy. This case is unique in that we report a new presentation of paediatric compartment syndrome and also highlight the importance of considering compartment syndrome as a differential for unexplained, increasing limb pain in this patient cohort.

## 2. Case Presentation

A 10-year-old boy with no past medical history presented with a 2-day history of central abdominal pain which subsequently migrated to the right iliac fossa. A diagnosis of acute appendicitis was made and an open appendicectomy was performed. There were no immediate postoperative complications and he was discharged on day 2 postoperatively. The patient represented to hospital 4 days later with increasingly severe left leg pain and inability to weight-bear. The leg was enlarged and erythematous. He denied any history of trauma. The patient was initially admitted under the medical team without consultation of the orthopaedic service, with a presumed diagnosis of a left sided below knee deep venous thrombosis.

Initial investigations revealed white cell count, C-reactive protein, and erythrocyte sedimentation rate within the normal range with a normal prothrombin and partial prothrombin time. Doppler ultrasonography of the lower leg arranged by the medical team revealed no evidence of deep venous thrombosis. The pain increased in intensity despite increasing dosage of opiate analgesia. The patient was referred to our orthopaedic service one day after admission. At this point the child had severe, unrelenting pain of his left lower leg. On examination his leg was hard throughout all compartments with hyperaesthesia of the anterior aspect of the leg. Severe pain was elicited on passive ankle plantar flexion, inversion, and dorsiflexion. Dorsalis pedis and posterior tibial artery pulses were palpable; however the leg was cold.

A diagnosis of acute compartment syndrome was made and he underwent emergent fasciotomy.

**Figure 1:**
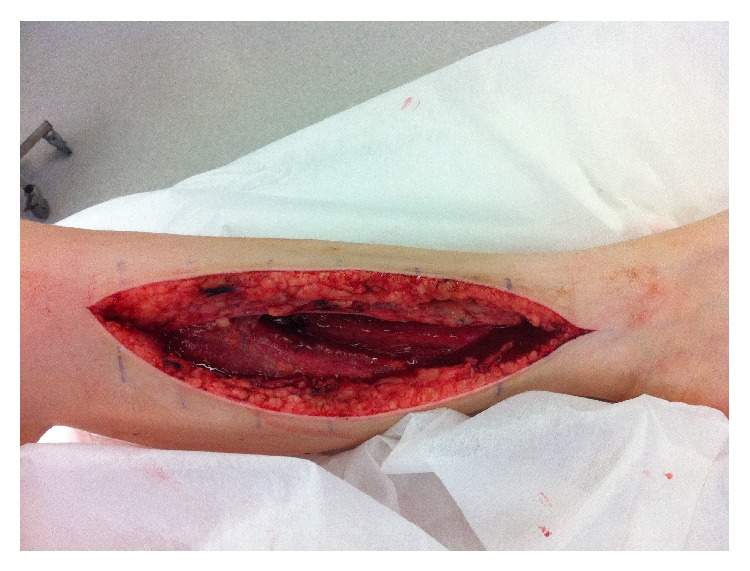
Medial fasciotomy of left lower leg.

**Figure 2:**
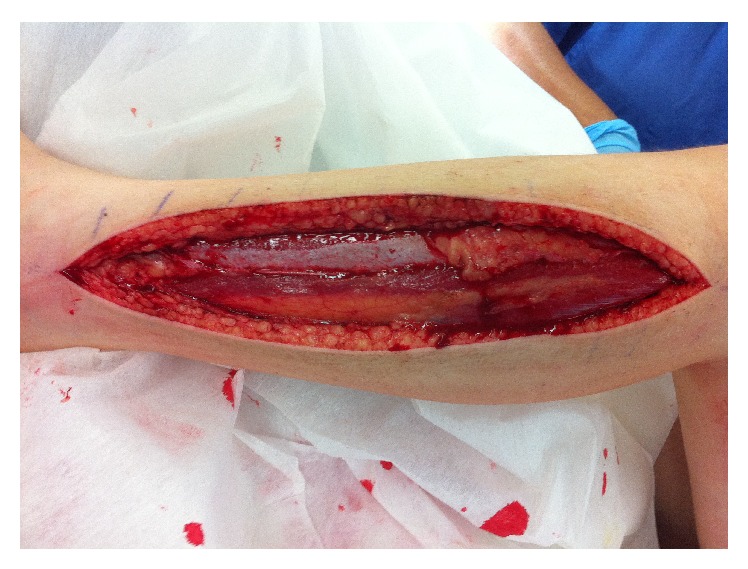
Lateral fasciotomy of left lower leg.

Immediate preoperative compartment pressure was measured using the Intracompartmental Pressure Monitor (Stryker). All four lower leg compartments had delta pressure readings (defined as diastolic blood pressure − intracompartmental pressure) less than 30 mmHg, which was diagnostic of compartment syndrome. A standard two-incision (medial and lateral) fasciotomy was performed decompressing all four compartments (Figures 1 and 2). The muscle tissue appeared viable at the initial fasciotomy and subsequent wound reviews revealed no evidence of necrotic tissue. The patient underwent three partial closure procedures before the fasciotomy wounds were definitively closed on day 12.

The patient made a slow recovery and had ongoing hyperalgesia in the affected area, despite thorough decompression of all four compartments. Repeated compartmental pressure monitoring over a one-week period revealed no evidence of residual compartment syndrome. The pain persisted for a further two months postoperatively and a diagnosis of chronic regional pain syndrome was made. He was treated by a multidisciplinary team (MDT) involving the orthopaedic team, physiotherapists, pain specialists, and psychologists. The pain improved with MDT management and he is now pain-free at 18-month follow-up with no residual functional or neurovascular deficit.

## 3. Discussion

Compartment syndrome in adults has been well described; however there is a relative paucity of information relating to the paediatric population [5]. Untreated it can progress from ischaemia to irreversible muscle and nerve damage. Therefore, early diagnosis and fasciotomy convey the best chance of recovery. To the best of our knowledge this is the first case of acute compartment syndrome of the leg in a child after appendicectomy.

The exact pathophysiological mechanism of compartment syndrome after appendicectomy in this case is unclear. However, there have been numerous descriptions of lower leg compartment syndrome occurring after general surgical, gynaecological, and urological procedures performed in the lithotomy position [6–8]. Penetrating abdominal trauma has also been associated with lower leg compartment syndrome [9]. It is hypothesised that ischaemia resulting from rising compartment pressures and decreased perfusion pressures in the lithotomy position may contribute to compartment syndrome [6]. Prolonged operative time has also been suggested as a contributory factor [6]. A similar mechanism of intraoperative hypoperfusion may have been involved in the case we describe; however a standard appendicectomy approach in the supine position was performed.

We feel the case is important as it highlights the necessity to maintain a high index of suspicion for compartment syndrome and early referral to the appropriate service. It also serves to highlight the atypical presentation and potential prolonged disability that can occur after compartment syndrome, even when adequately decompressed. This first description of compartment syndrome after appendicectomy reinforces the atypical presentation of the condition in children. In summary, any child presenting with increasing lower leg pain and increasing analgesic requirements with no apparent explanation should have an urgent orthopaedic referral to rule out compartment syndrome as the consequences of delayed diagnosis can be severe.
